# Ferrimagnetic Large Single Domain Iron Oxide Nanoparticles for Hyperthermia Applications

**DOI:** 10.3390/nano12030343

**Published:** 2022-01-21

**Authors:** Diana Zahn, Joachim Landers, Juliana Buchwald, Marco Diegel, Soma Salamon, Robert Müller, Moritz Köhler, Gernot Ecke, Heiko Wende, Silvio Dutz

**Affiliations:** 1 Institute of Biomedical Engineering and Informatics (BMTI), Technische Universität Ilmenau, D-98693 Ilmenau, Germany; diana.zahn@tu-ilmenau.de (D.Z.); juliana.buchwald@web.de (J.B.); 2 Faculty of Physics and Center for Nanointegration Duisburg-Essen (CENIDE), University of Duisburg-Essen, D-47057 Duisburg, Germany; joachim.landers@uni-due.de (J.L.); soma.salamon@uni-due.de (S.S.); heiko.wende@uni-due.de (H.W.); 3 Leibniz Institute of Photonic Technology (IPHT), D-07745 Jena, Germany; marco.diegel@leibniz-ipht.de (M.D.); robert.mueller@leibniz-ipht.de (R.M.); 4 Institute of Organic Chemistry and Macromolecular Chemistry (IOMC), Friedrich-Schiller-University Jena, Lessingstrasse 8, D-07743 Jena, Germany; moritz.koehler@uni-jena.de; 5 Institute for Micro- and Nanoelectronics, Technische Universität Ilmenau, D-98693 Ilmenau, Germany; gernot.ecke@tu-ilmenau.de

**Keywords:** hyperthermia, hysteresis, magnetic iron oxide nanoparticles, large single domain

## Abstract

This paper describes the preparation and obtained magnetic properties of large single domain iron oxide nanoparticles. Such ferrimagnetic particles are particularly interesting for diagnostic and therapeutic applications in medicine or (bio)technology. The particles were prepared by a modified oxidation method of non-magnetic precursors following the green rust synthesis and characterized regarding their structural and magnetic properties. For increasing preparation temperatures (5 to 85 °C), an increasing particle size in the range of 30 to 60 nm is observed. Magnetic measurements confirm a single domain ferrimagnetic behavior with a mean saturation magnetization of ca. 90 Am^2^/kg and a size-dependent coercivity in the range of 6 to 15 kA/m. The samples show a specific absorption rate (SAR) of up to 600 W/g, which is promising for magnetic hyperthermia application. For particle preparation temperatures above 45 °C, a non-magnetic impurity phase occurs besides the magnetic iron oxides that results in a reduced net saturation magnetization.

## 1. Introduction

Magnetic iron oxide nanoparticles (MNPs) are of great interest for hyperthermia applications as a therapeutic approach mainly for tumor diseases [1,2,3,4,5], utilizing their unique feature of heat generation when placed in an external alternating magnetic field caused by the reorientation of the magnetic moment of the particle. In this medical setting, strict limits have to be met concerning the applied magnetic field to ensure patient safety. For the product of field amplitude H and frequency f, Hergt and Dutz stated 5 × 10^9^ Am^−1^s^−1^ as a threshold [6]. This would, for example, restrict the field amplitude to 16 kA/m when a frequency of 300 kHz is used. As the required field amplitude for efficient re-orientation of the magnetic moment needs to be large enough compared with the coercivity (H_C_), the H_C_ of particles for medical applications is limited. As a rough guideline, field amplitudes should be two or three times the coercivity value [7]. Moving from direct medical applications to biotechnological or technical ones, safety limits are no longer needed, and stronger fields can be used. This enables the use of particles with higher coercivities and remanent magnetizations (M_R_) than the often-used small superparamagnetic MNP [8]. Technical hyperthermia applications can include thermoresponsive shape memory polymers with embedded MNPs, providing the needed temperature increase to enable the transition of the polymer from elastic to permanent shape [9,10,11].

Apart from heating applications, large ferrimagnetic MNPs can also be promising candidates for magnetic particle imaging (MPI) [12,13].

To prepare ferrimagnetic MNPs with a pronounced coercivity, several approaches can be utilized. Instead of using iron oxides as the core material, cobalt ferrites can be synthesized, where Fe^2+^ ions are replaced by Co^2+^ ions, leading to higher magnetic anisotropy [14] and good heating performance [15,16,17,18]. Anisotropy and thereby coercivity can also be increased by varying the shape of particles from spheres to rods, discs, or cubic structures [19,20,21,22,23,24].

In the present work, we synthesized large single domain iron oxide particles (LSDPs) with ferrimagnetic behavior. Increasing the diameter of spherical iron oxide particles enables larger coercivity values, as the energy barrier that needs to be overcome for magnetic reversal is a function of the particle volume. This coercivity increase reaches a maximum at the transition from single domain to multi domain particles. In multi domain particles, the orientation of the magnetic moment of the particles is changed by an external field due to Bloch wall movements in the crystal [25] and takes less energy than rotating a single domain of the same size [26,27].

Several groups investigated the influence of particle size on the magnetic behavior and heating performance of MNPs. Sung et al. synthesized magnetite nanoparticles in the size range of 10 to 500 nm with a transition from single to multi core around 120 nm, up to which the coercivity increased to values of 3.8 kA/m with increasing size and then decreased [26]. Ma et al. synthesized iron oxide MNP from 7.5 to 416 nm, also showing a clear maximum in coercivity around 46 nm, probably due to the evolution of several domains above this size [28]. Dutz et al. found the transition to multidomain iron oxide particles in the range from 70 to 80 nm [29]. Thermal decomposition was used by Tong et al. to synthesize highly uniform single domain particles up to 40 nm, which showed exceptionally high SAR values up to 2500 W/g for the largest particles [30].

Another strategy to synthesize large single domain particles is the oxidation method reported by Nishio et al., where so-called green rust precipitates from adding NaNO_3_ and NaOH to a solution of FeCl_3_ are formed and slowly becomes oxidized to magnetite under O_2_-free conditions [31]. This method was used by Li et al., resulting in particles with 24, 36, and 65 nm with H_C_ of 8.4, 11.1, and 15.1 kA/m, respectively; however, showing a decreasing SAR with increasing size and H_C_ [32]. The influence of reaction temperature on the resulting particles with this method was studied by Zhu et al. [33]. Increasing temperature from 23 to 30 °C decreases the particle size from 100 to 20 nm. The same trend was shown in the original publication of Nishio et al. [31] and Müller et al. reported maghemite LSDPs of 20.5 nm and a high coercivity of 11.2 kA/m using this method [34].

In the study presented here, we report on experiments about the influence of synthesis temperatures used for the Nishio method (5 to 85 °C) on the resulting particles’ structure and magnetism. The obtained LSDPs were characterized with TEM, X-ray diffraction, Auger electron spectroscopy, vibrating sample magnetometry, Mössbauer spectroscopy, and calorimetric hyperthermia measurements in agarose gels. The particles show increasing size and coercivity with increasing preparation temperature and the appearance of less magnetic Na-bearing parasitic phases above a synthesis temperature of 45 °C, as confirmed by Mössbauer spectroscopy and Auger electron spectroscopy, resulting in decreasing saturation magnetization. Highest SAR was measured for the sample synthesized at a medium temperature of 35 °C with more than 600 W/g for a frequency of 290 kHz and a magnetic field amplitude of 55 kA/m.

## 2. Materials and Methods

### 2.1. Preparation of Nanoparticles

The magnetic nanoparticles were prepared following a modified oxidation method, first published by Nishio et al. 2007 [31], but expanding the synthesis temperature range from 5 °C up to 85 °C. Correspondingly, the obtained samples are denoted here as “S05” to “S85”. To assure an oxygen-free reaction, all used solutions were flushed with N_2_ gas prior to the reaction, which was also continuously flushed with N_2_. Then, 10 mmol NaOH and 8.8 mmol NaNO_3_ were dissolved in 475 mL deionized and deaerated water, kept in a two-neck-flask, and heated to a distinct temperature (5 to 85 °C in 10 K steps) under permanent stirring in a water bath. Then, 25 mL of a deaerated 0.1 molar ferrous chloride solution was added to the flask and stirring continued for 24 h. No further reducing or stabilizing agents were used. The resulting particles were washed three times with deionized water and dried at room temperature for further use.

### 2.2. Characterization of Magnetic Nanoparticles

The obtained magnetic nanoparticles were characterized regarding their structural and magnetic properties as well as their magnetic heating performance, when exposed to an alternating magnetic field.

Transmission electron microscopy (TEM): For TEM images of the particles, copper grids were used after 2 min of cleaning with Ar plasma to assure a hydrophilic surface. Then, 10 µL of the particles suspended in water were applied to the grid and excess sample material was blotted with filter paper. Images were acquired using a 200 kV FEI Tecnai G2 20 (FEI, Hillsboro, OR, USA) equipped with a 4 k × 4 k Eagle HS CCD and a 1 k × 1 k Olympus MegaView camera for overview images.

X-ray diffraction (XRD): The crystal structure of the magnetic nanoparticles was investigated by means of XRD (Panalytical X’pert Pro, Malvern Panalytical, Almelo, The Netherlands). For this, MNP were measured in Bragg-Brentano geometry on spinning zero background holder (1 turn/sec) with a Cu source. The results of the XRD investigations provided information about the magnetic phase composition, the mean size of the MNP, as well as possible crystalline impurity phases. The mean size of the MNP was calculated from measurements of the XRD line width using the Scherrer formula.

Auger electron spectroscopy (AES): For Auger electron spectroscopy, by means of a microlab 350 (Thermo Fisher Scientific, Waltham, MA, USA), the powder samples were transferred to an ultra-high vacuum chamber. For analysis, they are bombarded by a 10 kV electron beam with a beam current of approximately 10 nA and 30 nm diameter. Using an SEM image, the spots or areas for measurement were chosen. The measured Auger electron spectra give information about the chemical composition only of the outermost atomic layers of the particles, when no ion beam sputtering is used. The information depth of this method depends on the Auger energy of the electrons and ranges from some atomic layers up to several nanometers.

Vibrating sample magnetometry (VSM): The magnetic characterization of the nanoparticle powder samples was carried out by utilizing the VSM option of a PPMS DynaCool (Quantum Design, San Diego, CA, USA), with field-dependent magnetization curves M(H) being recorded at 5 K and 300 K up to maximum magnetic fields of ±9 T. The field was ramped continuously, using a higher ramp rate (15 mT/s) in the high field region (±9 to ±0.5 T) and a lower rate (2 mT/s) in the low field region (±0.5 T) to ensure high point density. The measurements were performed on 5–10 mg of dry powder per sample, with the VSM capsules and sample holders supplied by the manufacturer ensuring a compact, cylindrical shape of the powder, as well as correct centering within the detection coils.

Mössbauer spectroscopy: Mössbauer spectra were recorded using ca. 20 mg of nanoparticle powder per sample in transmission geometry with a ^57^Co(Rh) radiation source mounted on a constant-acceleration driving unit. General characterization measurements were performed between 5 K and 300 K in a SHI-850-5 closed-cycle cryostat (Lake Shore Cryotronics, Westerville, OH, USA). Further analysis of the sample composition and magnetic alignment behavior was done via in-field Mössbauer spectroscopy in a liquid helium bath cryostat containing a superconducting magnet in split-pair geometry, providing a homogeneous magnetic field of 5 T along the γ-ray propagation direction.

Specific absorption rate (SAR): The determination of the SAR as a measure for the heating performance of the MNP when exposed to an alternating magnetic field was performed for immobilized particle samples, in order to simulate the in vivo behavior of the particles, which are not able to move freely once applied to biological tissue like tumors [35]. Therefore, a suspension of 1 wt% MNP in a 1 wt% agarose solution was prepared and distributed to three vials, each containing 0.5 mL of the particle-agarose-suspension. The samples were cooled down rapidly to avoid sedimentation of the particles during the hardening of the agarose gel and the sample mass was noted. Heating behavior of the samples was evaluated using an alternating magnetic field generator (SINAC 12 SH, EFD Induction GmbH, Freiburg im Breisgau, Germany) at a frequency of 290 kHz and at three different field amplitudes: 16, 27, and 55 kA/m, with a new measurement sample being used for each amplitude. Temperature curves were acquired using a fiber optic temperature probe (FOTEMP 2, Weidmann Technologies Deutschland GmbH, Dresden, Germany), placed in the center of the sample. Temperature curves were then used to calculate a temperature increase as a function of the heating time (∆T/∆t) and SAR was calculated using the following equation:

$$\mathrm{SAR}=\frac{∆T}{∆t}\frac{\;\cdot {\;m}_{S}\;\cdot \;c}{\cdot {\;m}_{\mathrm{MNP}}}$$

with total sample mass as m_S_, mass of MNP in the sample as m_MNP_, and the heat capacity of water c as 4.19 kJ/kgK [36].

## 3. Results and Discussion

For each synthesis, a black precipitate was obtained, which was air-dried to a powder at room temperature. As an intermediate, greenish precipitate was observed at the beginning of each synthesis with a slow transition to black throughout the reaction, we assume that green rust is formed as a first step and is further self-oxidized to magnetite. With the given educts, this presumed mechanism might be described with the following chemical sum formula:

$$12{\;\mathrm{Fe}}^{2+}+{\mathrm{NO}}_{3}{}^{-}+13{\;H}_{2}O\;\to 4{\;\mathrm{Fe}}_{3}O_{4}+23{\;H}^{+}+{\mathrm{NH}}_{3}\;$$

The total weight of the obtained dried particle powder was determined. With the used amounts of chemicals and assuming pure magnetite or maghemite as the resulting material, 0.19 and 0.20 g, respectively, is the maximum amount of nanoparticles that could be produced theoretically in one batch. 

**Figure 1:**
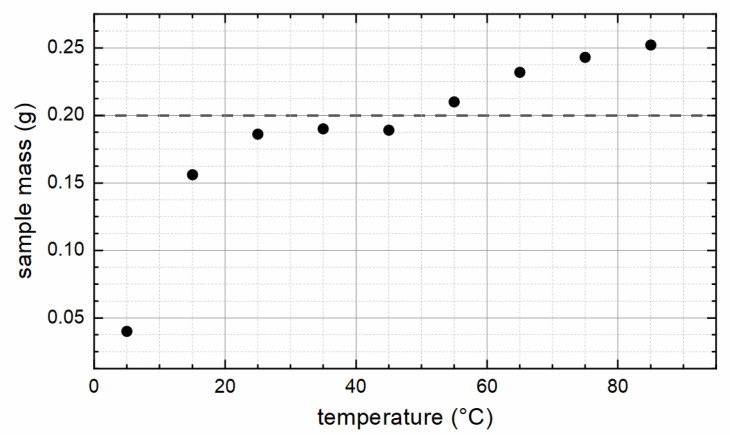
Total weight of the synthesized samples for each reaction temperature. The dashed line marks the maximum amount, if pure maghemite is formed.

As can be seen in Figure 1, with synthesis temperatures (for simplification referred to as “temperature” in all diagrams) above 50 °C, the weight of the reaction product exceeds this limit, which indicates the appearance of other phases apart from magnetic iron oxides magnetite and maghemite. The relatively low weight of the samples synthesized at 5 (S05) and 15 °C (S15) can be explained with the temperature being too low for a complete reaction in the given time of 24 h, so that the used educts only partially react to magnetic iron oxide nanoparticles.

**Figure 2:**
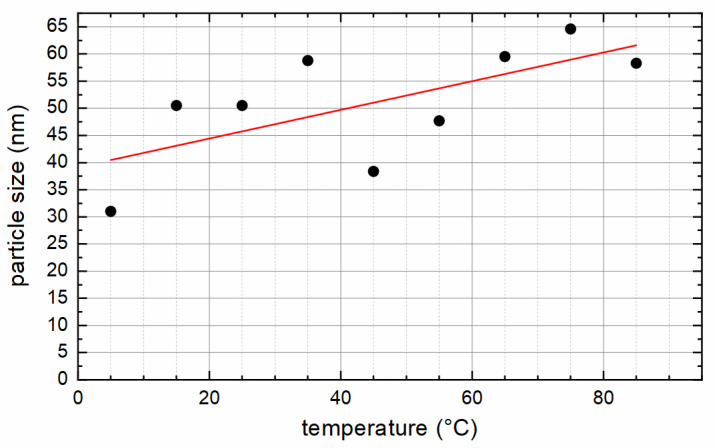
Particle diameter from XRD as a function of the synthesis temperature. Line serves as a guide to the eye only.

Table 1 summarizes the characterization results for all particles. The diameter of the particles, as measured by means of XRD, increases with increasing synthesis temperature (see also Figure 2), within a range of 30 to 65 nm, confirming that the chosen synthesis strategy enables the production of large magnetic nanoparticles that still exhibit a single core structure. XRD measurements also revealed only slight differences in the diffraction angle 2θ of the (440) peak, with 62.43° as the smallest value for S05 and 62.56° for S85, which are typical values for magnetite [37]. In the JCPDS database (file 19-0629), a value of 62.5711° is given for magnetite. A significant oxidation of magnetite to maghemite (γ-Fe_2_O_3_) can be excluded by XRD. The corresponding value of maghemite is 62.9811° (JCPDS file 39-1346). In XRD, no crystalline non-magnetic impurity phases within the samples were found.

**Figure 3:**
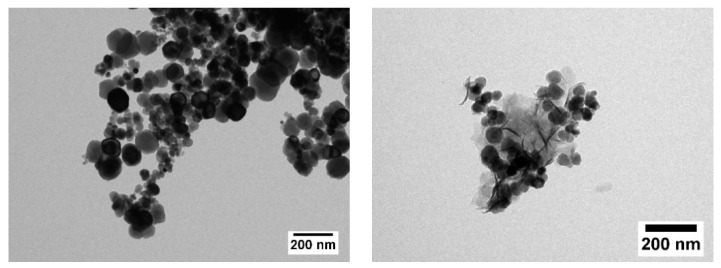
Typical TEM images for S35 (**left**) and S75 (**right**). An additional phase besides the MNP can be seen in S75.

Obtained TEM images revealed mainly spherically shaped particles with a rather broad size distribution of the LSDPs, which is expected for a wet chemical preparation route like the precipitation method used in this work. Owing to the limited colloidal stability of the samples, determination of size distribution by means of dynamic light scattering was not feasible. In TEM, we found no influence of synthesis temperature on the uniformity of the LSDPs, but concerning the particle shape, smaller particles are more sphere-like, whereas larger ones show a deviation from the spherical shape due to pronounced crystalline facets. Figure 3 shows particles synthesized at 35 °C (left) and the ones synthesized at 75 °C (right).

The appearance of other phases apart from magnetite and maghemite for reaction temperatures above 50 °C, as concluded from the obtained total sample mass, might also be seen in TEM images. For S75, needle-like structures can be seen in addition to the roughly spherical nanoparticles (see Figure 3 right), which might be iron hydroxides or salts.

Investigation into the sample composition by means of AES revealed for S35 the occurrence of Fe and O only, which confirms the iron oxide composition of pure magnetite or maghemite. In addition, smallest amounts of Si were detected, which can be attributed to the glassware used during synthesis. For S75, Na was found besides Fe and O with an approximate amount of 3–5 at%, estimated by the sensitivity factor method, which confirms an impurity phase within the samples obtained for reaction temperatures above 45 °C. The Na was detected in the outermost atomic layers of the particle grains, as no ion beam sputtering was applied. S75 also showed a different conductive behavior leading to static charges at the surface, which could be observed during the sample preparation for the measurements.

The composition of the samples was further investigated via magnetometry and Mössbauer spectroscopy, with the results shown below.

**Figure 4:**
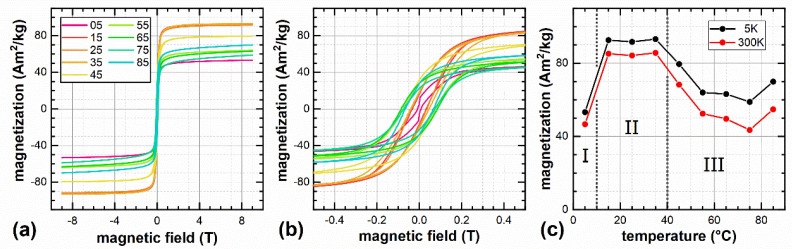
Magnetization of sample S05 to S85 recorded at 5 K in external magnetic fields up to 9 T (**a**) shown in magnification in (**b**). Maximum magnetization values recorded at 9 T at 5 K and 300 K, respectively (**c**), showing three distinct regions (I–III) with different magnetic behavior dependent on particle preparation temperature, as outlined in the text.

M(H) curves shown in Figure 4a,b display high magnetization close to bulk values for magnetite of ca. 98 Am^2^/kg [38] for sample S15 to S35 (region II in Figure 4c), with a maximum magnetization of 93.2 Am^2^/kg recorded at 5 K and 9 T for S35. In the high-field region, almost no further increase in M(H) is visible, wherefore the nanoparticles can be assumed to be nearly magnetically saturated. The minor reduction relative to bulk saturation magnetization can be assigned to a minimum degree of spin frustration at the particle surface as well as to the formation of a thin maghemite surface layer of slightly lower magnetization, both being verified in Mössbauer spectroscopy experiments as illustrated below. Samples prepared at higher temperatures (region III) exhibit lower high-field magnetization, which could be explained by the presence of materials of lower magnetization in addition to ferrimagnetic iron oxides, as discussed in more detail in the next section. S5 (region I) shows a similar decrease in M_S_ and a strongly deviating M(H) curve shape, which we assign to a mixture of iron oxide nanoparticles with unreacted precursor material or intermediaries of the nanoparticle synthesis owing to insufficient temperature.

**Figure 5:**
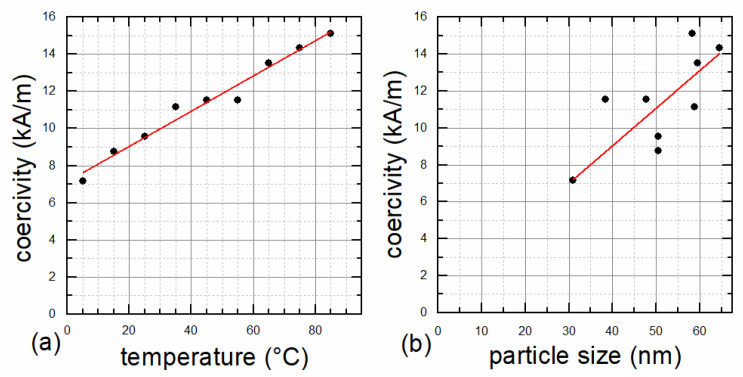
Coercivity at 300 K as function of synthesis temperature (**a**) and XRD particle diameter (**b**). Lines serve as a guide to the eye only.

VSM measurements also confirmed the single core structure, as the coercivity increases with increasing preparation temperature and essentially with an increasing particle diameter (see Figure 5), indicating that the transition from single to multicore structure is not yet reached. This transition would lead to decreasing coercivities with increasing particle size, after the peak for the largest possible single core particle was exceeded [7]. An almost linear correlation between coercivity and preparation temperature was found (Figure 5a), but stronger deviations for the correlation of coercivity and particle size can be seen in Figure 5b. This might be attributed to the fact that the method of determining the particles’ sizes by XRD does not work reliably for the MNP type used here. Furthermore, the coercivity of an MNP ensemble is determined by the mean particle size as well as the particle size distribution. The influence of the size distribution on the coercivity was not taken into account in Figure 5b. Measured coercivities are sufficiently high, ranging between 6 and 15 kA/m at 300 K, to achieve the desired ferrimagnetic behavior with an open hysteresis loop, suitable for hyperthermia applications. M_R_/M_S_ as an indicator for the squareness of the hysteresis curve ranges between 0.08 and 0.20 and shows good correlation with coercivity.

**Figure 6:**
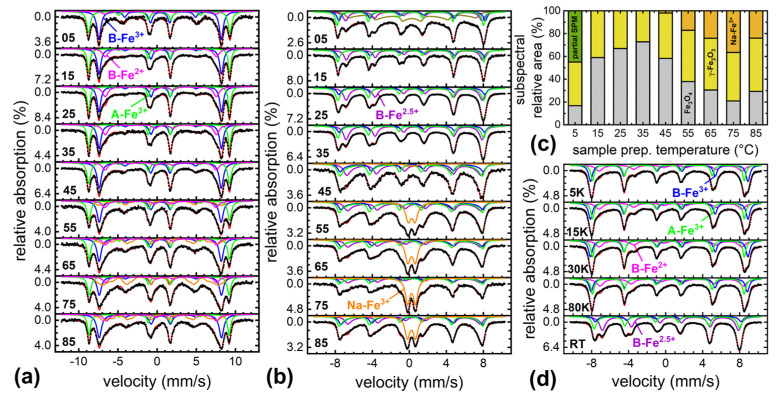
Mössbauer spectra of (**a**) samples S05 to S85 recorded at 4.3 K in an external magnetic field of 5 T parallel to the γ-ray propagation direction and (**b**) at room temperature. Relative subspectral areas representing sample composition as extracted from (**a**,**b**) are shown in (**c**). Mössbauer spectra of S35 recorded at 5–293 K are shown in (**d**) to demonstrate the magnetite Verwey transition. Subspectra include B-site Fe^3+^ (blue), A-site Fe^3+^ (green), B-site Fe^2+^ (violet), B-site Fe^2.5+^ mixed valence states (purple), and a hyperfine field distribution/doublet assigned to the parasitic Na-bearing Fe^3+^-phase (orange). At room temperature, for S05, a broad hyperfine field distribution (olive, (**b**)) is observed, presumably originating from beginning superparamagnetic relaxation (SPM) [42].

For a more detailed analysis of the nanoparticles’ magnetic structure and composition, extensive studies of the sample material were performed via Mössbauer spectroscopy upon variation of sample temperature and magnetic field, as displayed in Figure 6. For all samples, primarily subspectra characteristic of magnetite (Fe_3_O_4_)–maghemite (γ-Fe_2_O_3_) mixtures were identified. At 4.3 K (Figure 6a), these include sextets corresponding to Fe^3+^ in octahedral B-site coordination (blue) and tetrahedral A-site coordination (green) for maghemite and magnetite as well as a smaller contribution of B-site Fe^2+^ for magnetite only (violet) [39]. By comparing the relative spectral area of B-Fe^2+^ to ca. 33% as expected in pure magnetite, we can estimate the magnetite to maghemite ratio, yielding ca. 60–70% of Fe_3_O_4_ for sample S15–S35. The samples containing a higher fraction of magnetite as compared with maghemite are consistent with XRD data evaluation. This composition most likely corresponds to a particle structure composed of a magnetite core and an oxidized maghemite passivation surface layer [40] of ca. 3 nm in thickness. To check the sample stability, a second series of Mössbauer spectra was recorded 8 weeks after the first one, with the samples being stored under ambient conditions, showing further oxidation from magnetite to maghemite by only ca. 5% (data not shown), verifying the nanoparticles’ long-term stability. In addition to sample composition, in-field Mössbauer spectroscopy allows for the analysis of the degree of spin frustration, based on the relative intensity of lines 2 and 5. For S15 to S35, very moderate spin frustration with average spin canting angles of ca. 16 ± 1° is visible, which translates to approximately 96% of saturation magnetization at 5 T [41].

For S45–S85 (region III), an additional broad subspectral distribution arises (orange in Figure 6a), reaching a maximum relative area for S75. Owing to superposition with B-Fe^3+^ and B-Fe^2+^ subspectra, the precise hyperfine parameters are difficult to extract. However, the isomer shift of ca. 0.5 mm/s and the high contribution to lines 2 and 5 indicate an Fe^3+^-bearing antiferromagnetic material. Comparing measurements at 4.3 K to room temperature spectra in Figure 6b, we observe a transition of this component into a doublet subspectrum with a rather abrupt change in spectral structure visible in T-dependent measurements, pointing to a phase transition rather than to beginning superparamagnetic relaxation (not shown in detail). The presence of an antiferromagnetic phase in the nanoparticular samples could explain the reduction in high field magnetization in region III, precisely matching the relative amounts of this parasitic phase estimated from Mössbauer spectroscopy (Figure 6c, orange); a maximum relative area of ca. 37% of this parasitic phase is present for S75, also showing the lowest magnetization. It is reasonable to assume that this spectral component visible in region III corresponds to acicular shaped particles, which stood out in TEM images (see Figure 3) of S45 to S85, presumably bearing the Na-fraction detected in Auger spectroscopy.

Series of reference spectra were recorded for each sample between 5 K and 293 K, not only showing the sextet to doublet transition of the parasitic phase mentioned above, but also exemplarily of the magnetite Verwey transition for S35. The latter results in the combination of B-site Fe^3+^ and Fe^2+^ subspectra into a mixed valence Fe^2.5+^ state (purple), often explained by fast electron hopping between different octahedral positions [43].

As discussed above, the reduction in magnetization of S45–S85 (region III) as compared with region II was explained by the presence of a parasitic antiferromagnetic Na-bearing Fe^3+^ byphase. Nevertheless, a decreased high-field magnetization was also observed for S05 (region I), although no additional subspectral contribution was present in low temperature in-field spectra. While the decrease in M_S_ could stem from the slightly lower average particle diameter of ca. 30 nm, also leading to beginning superparamagnetic relaxation in room temperature spectra (Figure 6b, S05, olive), the distinct decrease in magnetization more likely indicates remaining Fe-free precursor material not visible in Mössbauer spectroscopy, which did not fully react owing to the limited reaction temperature.

**Figure 7:**
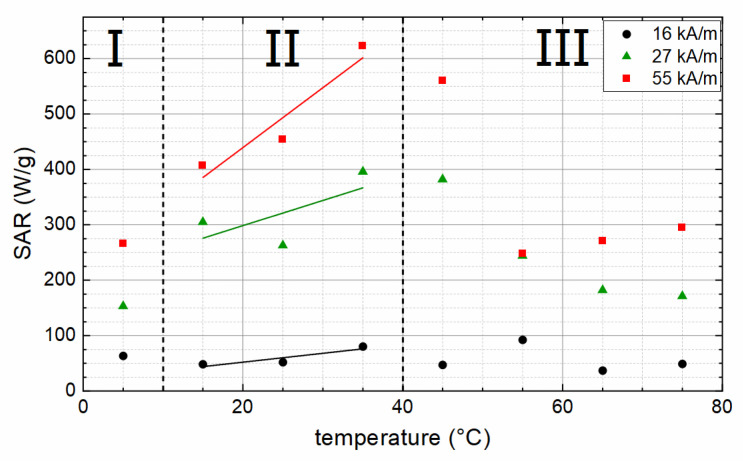
SAR as function of the particle synthesis temperature, measured at 16, 27, and 55 kA/m, showing three distinct regions (I–III) with different magnetic behavior dependent on particle preparation temperature, as outlined in the text. Lines serve as a guide to the eye only.

SAR was measured for immobilized particles at three different field amplitudes: 16, 27, and 55 kA/m. Because, for sample S85, no homogeneous dispersion of the particles within the agarose gel matrix could be achieved (probably because of a too high impurity phase content), this sample was excluded from SAR investigation. As can be seen in Figure 7, higher field strength leads to higher SAR values, indicating that, with the used field strengths, the saturation, where higher field strength does not lead to increasing SAR anymore, is not yet reached. Additionally, the lowest used field strength of 16 kA/m generates rather low SAR values, ranging from 37 to 92 W/g_MNP_ and not showing any correlation with particle properties like size or coercivity. Looking at the coercivity values of the particles of 6 to 17 kA/m, one can assume that 16 kA/m is insufficient to enable rotation of the magnetic moment of the particles and, thereby, generate heat efficiently. As a rough estimation, field strengths double or threefold the coercivity of the particles are needed for sufficient heating performance of magnetic particles [7]. The exact value for this factor depends on the width of the switching field distribution. For broad distributions with a mean value much larger than H_C_, the needed factor might be even larger [34].

For 27 and 55 kA/m, SAR values correlate to the magnetic properties of the particles. In region I, the particles show a rather low M_S_ (see Figure 4c), leading to low SAR values as magnetization is one of the key parameters for the size of the hysteresis curve and, thereby, the possible amount of heat that can be generated by the particles. In region II, SAR values increase with the increasing temperature, correlating with the increasing coercivity of the particles (coercivity values pictured in Figure 5). Contrary to the increasing H_C_, SAR values then decrease above a synthesis temperature of 45 °C (region III), which can be explained with the decreasing M_S_ (compare Figure 4c) and the presence of non-ferrimagnetic material in the particles, which diminishes the amount of heat that can be generated via hysteresis losses. The highest SAR of 622 W/g_MNP_ is measured for S35, which also shows a high M_S_ of 85.7 Am²/kg.

**Figure 8:**
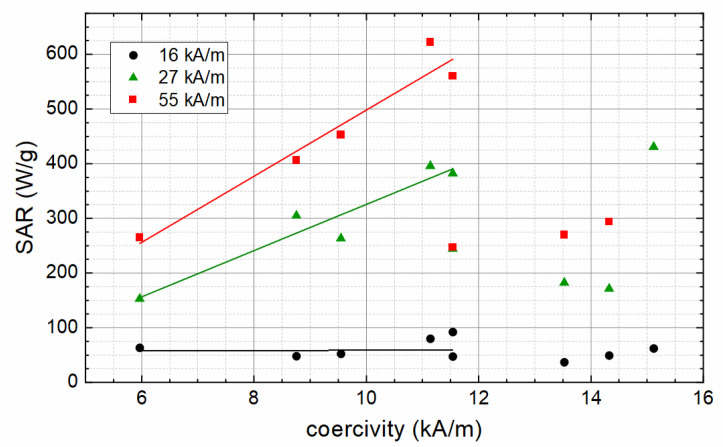
SAR values in dependence of coercivity. Lines serve as a guide to the eye only.

The same influence of M_S_ on SAR as discussed above can be found when the SAR is shown as a function of coercivity (see Figure 8). Up to a coercivity of about 12 kA/m, an increasing coercivity leads to increasing SAR. This can be related to the increasing coercivity of larger particles, leading to a larger area of hysteresis loop and, thereby, higher hysteresis losses when reversing the magnetization of the particles. Samples with coercivities above 12 kA/m originate from preparation temperatures above 45 °C and show a notable decrease in saturation magnetization, which results in lower SAR values.

As measurements to determine the SAR were performed at a particle concentration of 10 mg/mL, which is similar to the concentration for hyperthermia treatment, a raw estimation of the reachable temperature increase during hyperthermia can be provided. 

For a field strength of 27 kA/m (which is conceivable for medical hyperthermia), S35 shows an SAR of 400 W/g. Starting from room temperature (20 °C), this sample reaches 43 °C after 19 s and 70 °C after 50 s. For the maximum field strength of 55 kA/m used here, S35 with an SAR of 622 W/g reaches 43 °C after 13 s and 100 °C after 50 s, starting from 20 °C. Concluding from these values, the LSDP prepared here seem to be very promising for medical hyperthermia applications.

## 4. Conclusions

In this study, magnetic large single domain iron oxide nanoparticles were prepared by a modified oxidation method of non-magnetic precursors. The particle size was adjusted by variation of the temperature during the preparation of the non-magnetic precursors. From the synthesis, magnetic nanoparticles of mainly magnetite were obtained, showing an increasing diameter for increasing preparation temperatures, revealed by XRD measurements. The higher particle size results in an increasing coercivity and remanence and a single domain ferrimagnetic behavior was confirmed. A saturation magnetization of the particles of about 90 Am^2^/kg confirms the predominant magnetite phase, which is also verified in Mössbauer spectroscopy experiments. For preparation temperatures above 45 °C, an additional non-magnetic impurity phase occurs in the samples besides magnetite. XRD revealed no information about the nature of the impurity phase. By means of AES, a small proportion of Na was found in the samples prepared at higher temperatures. This could correspond to needle-shaped particles observed in TEM, a decreasing saturation magnetization, and the presence of an additional Fe^3+^ (paramagnetic) subspectrum in room temperature Mössbauer spectra, all being visible only for preparation temperatures above 45 °C. For the plain magnetite particles, an SAR promising for magnetic heating application occurs, and in good agreement with the theory, larger particles with higher coercivity show a higher heating performance. As soon as the particles contain a non-magnetic impurity phase, and thus a lower net saturation magnetization, the SAR decreases significantly, despite those particles showing the higher coercivity values (paired with a sufficiently high field amplitude) and allowing a high SAR to be expected. In ongoing work, we will investigate the non-magnetic impurity phase in more detail and evaluate strategies to prevent the formation of the non-magnetic phase during the preparation.

## Figures and Tables

**Table 1:** XRD and VSM results for all particles.

Sample	XRD		VSM at 300 K/9 T			VSM at 5 K/9 T		
T	D	2theta	Ms	Hc	Mr/Ms	Ms	Hc	Mr/Ms
(°C)	(nm)	(°)	(Am²/kg)	(kA/m)		(Am²/kg)	(kA/m)	
05	31.0	62.43	46.7	6.0	0.08	53.3	7.2	0.12
15	50.5	62.53	85.2	8.8	0.08	92.6	26.3	0.18
25	50.5	62.51	84.2	9.6	0.10	91.7	29.4	0.19
35	58.8	62.50	85.7	11.1	0.11	93.2	39.8	0.29
45	38.4	62.49	68.3	11.5	0.13	79.5	53.3	0.40
55	47.7	62.48	52.5	11.5	0.15	64.0	60.5	0.44
65	59.5	62.50	49.6	13.5	0.16	63.2	67.6	0.44
75	64.6	62.55	43.5	14.3	0.16	58.9	58.1	0.39
85	58.3	62.56	54.8	15.1	0.20	69.9	52.5	0.40

## Data Availability

The data presented in this study are available in the article.
